# Supplementary material for: Synergistic Upregulation of Target Genes by TET1 and VP64 in the dCas9–SunTag Platform
Source: Int J Mol Sci. 2020 Feb 25;21(5):1574. doi: 10.3390/ijms21051574 (PMC7084704; doi:10.3390/ijms21051574)
Supplement: Supplementary file 1 [file ijms-21-01574-s001.pdf]

Table S1

Target gene	Target sgRNA name	Target sequence	Primer name	Primer sequence
CARD9	gRNA-CARD9_3	TGGGAGCAGCTTTCCTCTGG	CARD9-1	CAGGCTCCTGGTGTGTCTG
			CARD9-2	CTCCAGCACTCGTCATCGT
KDM2B	gRNA-KDM2B_1	GGACTGCAAAGGCTCTGGTC	KDM2B-1	CATGCAGCAGAAAAGCAAAA
			KDM2B-2	TCCGACAAGTCCTCGTTCTC
RAB19	gRNA-RAB19_1	GCCTCAGGGTACAAAGCGCC	RAB19-1	GCTGAAAAGCAAACCCAGAA
			RAB19-2	AGCTGGAGAAGTGCATGGTT
CNKSR1	gRNA-CNKSR1_1	TGTGAGCCCAGGTATGCAGT	CNKSR1-1	GGCAAAACAGGAGCTGATTC
			CNKSR1-2	TAGTCCTGCAGGGAGTCGTC
SBNO2	gRNA-SBNO2_1	GGTAATTGCCCTGACTTACG	SBNO2-1	GCAGTTTGAGGCTCTGAACA
			SBNO2-2	ATGGTGGACACCTGGTTGAG
SPARC	gRNA-SPAARC_1	GAAGGGCCAAGCAATTCAAG	SPRC-1	GGTTTCCTGTTGCCTGTCTC
			SPRC-2	AAGAAGATCCAGGCCCTCAT
CLEC11A	gRNA-CLEC11A_1	ATGGTTCTGAGCGGGTTCCT	CLEC11A-1	GAGAGGGAGGCCCTGATG
			CLEC11A-2	GTCCTCCTCCATCTCCCAGT
HGF	gRNA-HGF_1	GCTCCTATCCGAGTAAGGAA	HGF-1	CATGTCCTCCTGCATCTCCT
			HGF-2	TGCTGGATCTATTTTGATTAGGG
TCF21	gRNA-TCF21_4	CGGGAGGCCTCTTGGTTTCA	TCF21-1	TGAGGCAGATCCTGGCTAAC
			TCF21-2	TCAGGTCACTCTCGGGTTTC
TINAGL1	gRNA-TINAGL1_1	CCTGCTTGGGGTTCCCCTAA	TINAGL1-1	CAGCCCACACCTTCACCA
			TINAGL1-2	GGCACCCAGAGCCAAGTG

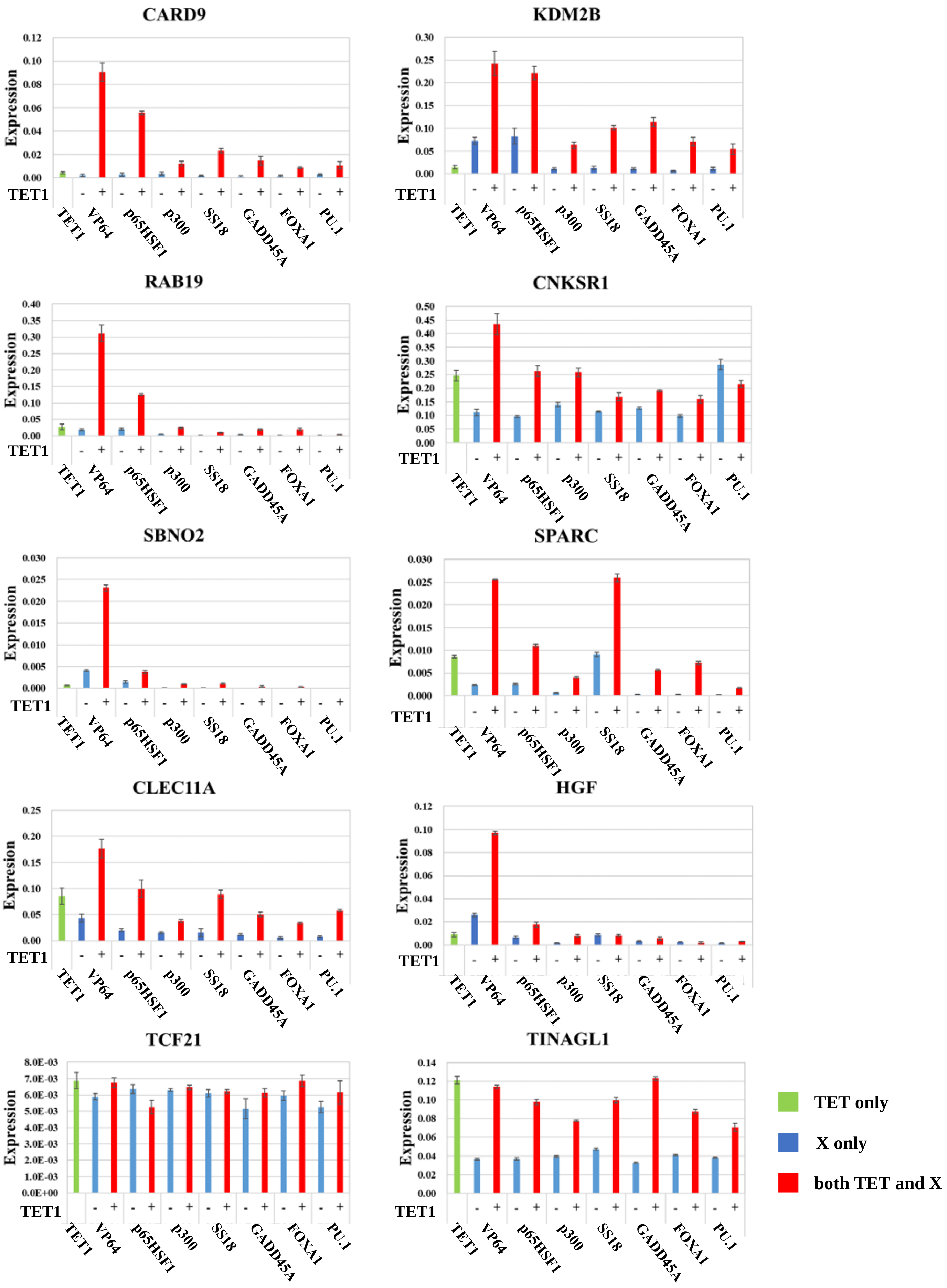
CARD9
KDM2B
RAB19
CNKSR1
SBNO2
SPARC
CLEC11A
HGF
TCF21
TINAGL1
Expression
TET1
- +
TET1
VP64
p65HSF1
p300
SS18
GADD45A
FOXA1
PU.1
TET only
X only
both TET and X

Supplementary Figure 1. Expression levels (not presented as fold changes to GFP-transfected experimental controls) in A549 cells transfected with dCas9–SunTag and scFv–TET1 (green bar); dCas9–SunTag and scFv–X (blue bar); and dCas9–SunTag, scFv–TET1, and scFv–X (red bar). Expression levels, determined by RT-PCR, were normalized against the corresponding level of actin mRNA. Results obtained using sgRNAs targeting *CARD9, KDM2B, RAB19, CNKSR1, SBNO2, SPARC, CLEC11A, HGF, TCF21*, and *TINAGL1* are shown.
